# Nicotinamide Adenine Dinucleotide Phosphate Oxidases and Metabolic Dysfunction-Associated Steatotic Liver Disease

**DOI:** 10.3390/antiox14010083

**Published:** 2025-01-13

**Authors:** Vittoria Cammisotto, Emanuele Valeriani, Pasquale Pignatelli, Francesco Violi

**Affiliations:** 1 Department of Clinical Internal, Anesthesiological and Cardiovascular Sciences, Sapienza University of Rome, 00185 Rome, Italy; vittoria.cammisotto@uniroma1.it (V.C.); pasquale.pignatelli@uniroma1.it (P.P.); francesco.violi@uniroma1.it (F.V.); 2 Department of General Surgery and Surgical Specialty, Sapienza University of Rome, 00185 Rome, Italy; 3 Department of Infectious Disease, Azienda Ospedaliero-Universitaria Policlinico Umberto I, 00161 Rome, Italy

**Keywords:** MASLD, NADPH oxidase, oxidative stress, liver disease

## Abstract

Metabolic dysfunction-associated steatotic liver disease (MASLD) is characterized by lipid accumulation in the liver due to an excess in their supplies or an impairment in their management. While some patients remain stable for years, a proportion of them progress up to steatohepatitis (MASH). MASLD links with systemic pathways being associated with metabolic and non-metabolic diseases. Although liver lipid accumulation represents the first hit for MASLD, the pathophysiology of its development and progression to MASH remains not completely understood. Oxidative stress has received particular attention in recent years, as most of the oxidative process occurs in the liver, which is also the target of oxidative stress-induced damage. Growing evidence linked the activity of nicotinamide adenine dinucleotide phosphate (NADPH) oxidases (NOX) to the increased liver production of reactive oxygen species up to liver damage and fibrosis. NOX acts both in hepatocytes and in non-parenchymal hepatic cells, contributing to hepatocyte lipotoxicity, impaired hepatic microcirculation, hepatic stellate, and mesenchymal stem cells activation and proliferation. This review aims to summarize the current knowledge on the involvement of oxidative stress in the MASLD–MASH transition, focusing on the role of NOX isoforms, and to suggest targeting NOX as a therapeutic approach in MASLD.

## 1. Introduction

The terms metabolic dysfunction-associated steatotic liver disease (MASLD) and steatohepatitis (MASH) have been recently introduced in clinical practice, taking the place of previous definitions (e.g., non-alcoholic fatty liver disease—NAFLD—, non-alcoholic steatohepatitis—NASH—) that did not correctly report the etiology of the disease and its link with systemic pathways [1,2]. These conditions, indeed, have been associated with several clinical entities comprehending other metabolic comorbidities (e.g., impaired insulin response, type 2 diabetes mellitus, dyslipidemia, hypertriglyceridemia, arterial hypertension) or chronic diseases (e.g., chronic kidney failure), cardiovascular diseases and mortality, malignancy [3,4]. More importantly, the prevalence of MASLD, as well as that of overweight and obesity, has increased dramatically in recent years (up to roughly 40%) being one of the most common liver disorders worldwide and requiring clinical and therapeutic attention [5,6].

The clinical course of MASLD is highly variable and is actually poorly predictable, ranging from asymptomatic alterations that maintain for several years up to decompensated liver cirrhosis [7]. A non-negligible proportion of MASLD patients (up to 50%) remains stable for several years, and an improvement has been reported in about 10% of patients [8]. Conversely, it has been estimated that the rate of fibrosis progression is lower in patients with MASLD than in patients with MASH—1 stage of progression every 14.3 years versus 7.1 years—and that up to 60% of patients with MASLD may progress to MASH [9,10]. Even if these progressions are promoted by both environmental and genetic factors, the precise underlying mechanisms remain partly understood [2]. Among the others, oxidative stress gained particular attention in MASLD and MASH development during the last years as most oxidative processes occur in the liver, which is also the target of oxidative stress-induced damage [11].

By definition, oxidative stress is the result of an imbalance between the production and the removal of reactive oxygen species (ROS). ROS are highly reactive molecules with unpaired electrons in their outer orbital, which initiate chain reactions and lead to irreversible chemical changes in lipids, proteins, and other macromolecules. ROS are by-products of normal cellular activity. They are produced in many cellular compartments and play an important role in signaling pathways. Their production generally occurs through the up-regulation (e.g., nicotinamide adenine dinucleotide phosphate—NADPH—oxidase—NOX—) or down-regulation (e.g., superoxide dismutase and glutathione peroxidase) of specific enzymes [11,12]. Being highly toxic molecules, ROS cause cellular dysfunction, damage, and death and lead to a chronic inflammatory condition that may enhance liver disease and cause the progression of liver damage [11].

## 2. Pathophysiology of MASLD and MASH

A large body of evidence trying to identify the underlying mechanisms of MASLD development has been published in the last few years. However, its pathophysiology is still not completely understood and a complex process including two hits has been first postulated, with the first hit representing lipids accumulation in the liver and the second one their oxidative stress-related alterations [13]. Further molecular pathways (e.g., genetically determined alterations) with different types of expression among patients have been recently identified, extending the previous definition [14].

### 2.1. MASLD and MASH Development

The pathogenetic mechanism of the so-called liver steatosis of MASLD is characterized by lipid accumulation in the liver due to an excess in their supplies or an impairment in their management [14,15]. Lipoproteins are heterogeneous groups of compounds characterized by a monolayer of membrane lipids—mostly phosphatidylcholine and unesterified cholesterol—and a hydrophobic lipoprotein core that contains triglycerides, cholesterol esters and lipophilic A, D, E, and K vitamins [15]. Lipoproteins vary in terms of size, lipid composition, and apolipoprotein content, are synthesized in different tissues, and are responsible for different roles in systemic lipid metabolism [15]. Once the triglyceride-rich lipoproteins give their contents to specific organs for energy production, the so-called remnant lipoproteins are taken up by hepatocytes through receptor-mediated endocytosis [15]. In this physiologic scenario, the impaired lipolysis of triglycerides from insulin-resistant adipose tissue represents a major driver of fatty acid accumulation into the liver from the blood and is a key factor for MASLD development [16,17,18,19]. In a study on 229 obese patients, the metabolic parameters (e.g., plasma insulin, plasma free fatty acid), hepatic insulin resistance, and liver fibrosis of those with MASLD worsened according to the quartiles of adipose tissue insulin resistance [17]. Inappropriate diet and weight gain surely contribute to this phenomenon as it has been reported that even a modest weight reduction (5%) provides a beneficial effect on adipose tissue, liver, and skeletal muscle insulin resistance without a concomitant change in systemic or subcutaneous adipose tissue markers of inflammation [20].

De novo lipogenesis from glucose and fructose and a reduced diversion of energy substrates to skeletal muscle or peripheral and brown adipose tissues also contribute to lipid accumulation in the liver [14,21,22,23]. A recent study highlighted that even a short-term fructose dietary restriction (nine days) reduced liver fat, interfering with de novo lipogenesis [24]. Similarly, the use of peroxisome proliferator-activated receptor gamma (e.g., pioglitazone) improved MASLD and MASH, enhancing fat storage in adipose tissue instead of in the liver [22]. A further interesting finding is the brown adipose and skeletal muscle tissues thermogenesis upregulation by bile acids through the activation of the Takeda G-protein-coupled receptor 5 [23]. The diversion of energy substrates to these tissues through specific therapies acting on bile acids may interfere with MASLD development [25,26].

Ultimately, fatty acid accumulation leads to increased production of triglycerides, which, in part, remain in the liver and constitute lipid droplets as a part of an adaptive and protective mechanism in cases of a higher fatty acid supply compared to the capacity to metabolize them [14]. This latter process is generally benign as inert lipid species accumulation (e.g., triglycerides and cholesterol esters) prevails. In steatohepatitis, there are high concentrations of other lipid species (e.g., free cholesterol, diacylglycerols, and ceramides) that can promote inflammatory responses up to cell death and fibrosis—a process named lipotoxicity—through alterations in endoplasmic reticulum and mitochondria, in intracellular signaling pathways, and in specific proinflammatory cellular kinases of the cell surface or cytoplasm [27,28,29,30]. In this setting, an overproduction of ROS contributes to chronic inflammation, leading to a disruption of the oxidant–antioxidant balance [29,30]. Similarly, it has been recently demonstrated that lipopolysaccharide is localized in hepatocytes and may contribute to liver inflammation through a Toll-like receptor 4 positive macrophages and platelets [31]. Higher levels of lipopolysaccharide and Toll-like receptor 4 positive macrophages and platelets were found in MASLD patients than controls and positively correlated with serum lipopolysaccharide levels [31].

### 2.2. Oxidative Stress in MASLD and MASH

Oxidative stress occurs when the production of ROS exceeds the capability of antioxidant pathways to neutralize them. ROS are oxygen-containing molecules with high chemical reactivity and comprehend both free radicals (e.g., hydroxyl, superoxide, peroxyl radicals) and nonradicals (e.g., hydrogen and lipid peroxides) [12]. ROS damage to lipids results in the formation of specific products such as malondialdehyde, lipid peroxide, 8-isoprostane, and 4-hydroxy-2-nonenal [32]. Being responsible for ROS production through its metabolic and detoxification activities, the liver is also one of the most exposed organs to ROS-related damage [33]. Hepatocytes represent a relevant site of ROS production, mostly in their mitochondria, as well as ROS production induces, in turn, mitochondria dysfunction in hepatocytes [33]. ROS also promote hepatic stellate cell activation and differentiation, leading to collagen and extracellular matrix compound accumulation in the liver [33]. Finally, Kupffer cells produce ROS in response to specific stimuli with a relevant impact on liver damage [33].

The sources of ROS in MASLD and MASH include electron leakage during mitochondrial metabolism (β-oxidation), peroxisomal β-oxidation, inhibition of mitochondrial electron transport chain, increased microsomal cytochrome p450 enzymes activity, endoplasmic reticulum stress response, increased xanthine oxidase activity, and abnormal inflammatory response [32,34,35]. Several biomarkers of oxidative stress have been identified during the last few years in patients with MASLD and MASH as well as reduced liver concentration/activity of both enzymatic (e.g., catalase, superoxide dismutase, glutathione peroxidase, glutathione reductase) and non-enzymatic antioxidants (e.g., ascorbic acid, glutathione, α-tocopherol, ubiquinone, thioredoxin, and bilirubin) has been found in clinical and experimental models of MASLD and MASH [32]. Conversely, increased liver or serum and plasmatic levels of oxidative stress biomarkers (e.g., nitric oxide, malondialdehyde, 8-hydroxy-2′-deoxyguanosine, and cytochrome P4502E1) have been found in clinical and experimental models [32].

### 2.3. NOX-Related ROS Production in the Liver

**Figure 1:**
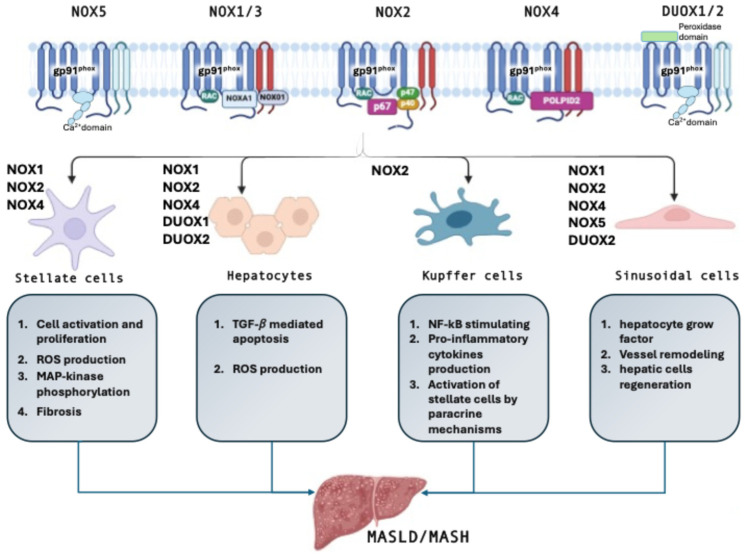
Isoforms of the NOX family in the liver. Liver cells, such as hepatic stellate cells, hepatocytes, Kupffer cells, and sinusoidal endothelial cells, express different NOX isoforms that share a structural homology based on a common catalytic core consisting of six transmembrane helices, known as the gp91phox. These NOXs contribute to MASLD and MASH development, by acting through multiple pathways. MASH, metabolic dysfunction-associated steatohepatitis; MASLD, metabolic dysfunction-associated steatotic liver disease; NOX, nicotinamide adenine dinucleotide phosphate oxidases.

NOX is an enzyme system constituted by a multi-component complex of proteins (Figure 1). Seven NOX isoforms have been identified, including NOX1, NOX2, NOX3, NOX4, NOX5, and dual oxidases (DUOX) 1 and 2 [36]. Interestingly, NOX2, NOX3, NOX4, DUOX1, and DUOX2 are present in several mammalian species. However, some mammalian species have lost NOX1, others have lost NOX5, and NOX5 is absent in mice and rats [37,38]. All NOX isoforms are membrane-bound enzymes that rely on NADPH for their activity, and the major source of ROS is generated when the flavin- and haem-containing protein complex transfers electrons from cytosolic NADPH to molecular oxygen to produce O^2−^ or H_2_O_2_ [39,40]. This latter mechanism regulates several redox-sensitive pathways [41]. Conserved domains have been identified in the specific isoforms of NOX. All NOX isoforms share structural homology based on a common catalytic core composed of six transmembrane helices chelating two hemes and a dehydrogenase domain binding the non-covalently bound flavin cofactor and the NADPH substrate. However, they differ in cellular and tissue distribution, activation mechanism, or regulatory system [42].

Growing evidence linked the activity of NOX to the increased liver production of ROS—mostly superoxide (O_2_^−^), hydrogen peroxide (H_2_O_2_), and hydroxyl radical (OH)—up to liver damage and fibrosis [43,44]. It has been further demonstrated that NOX acts both in hepatocytes and in non-parenchymal hepatic cells (e.g., hepatic stellate cells, macrophages—Kupffer cells—, lymphocytes, and liver sinusoidal endothelial cells). NOX contributes to hepatocyte lipotoxicity through mitochondrial oxidative phosphorylation dysfunction [45]. Furthermore, it reduces the bioavailability of nitric oxide to cause an impaired hepatic microcirculation and to enhance the peroxynitrite-induced hepatocellular injury [46]. Through the activation of specific pathways, NOX also directly stimulates the directional migration of hepatic stellate cells and mesenchymal stem cells as well as their activation, proliferation, and collagen production [47,48,49,50,51,52]. The reduced hepatic stellate cell activation following antioxidant supplementation (e.g., ascorbic acid or α-lipoic acid) confirms these data [53,54].

## 3. NOX in the Liver

The seven members of the NOX family vary in tissue expression levels and activation mechanisms. Several NOX isoforms are expressed by both parenchymal and non-parenchymal liver cells as well as the expression of NOX isoforms vary among different types of liver cells (Figure 1) [55,56]. The NOX isoforms of main interest in the liver are the NOX1, NOX2, and NOX4. While these latter are expressed by both hepatocytes and hepatic stellate cells, Kupffer cells mainly express NOX2 [57].

### 3.1. Hepatic Stellate Cells

Hepatic stellate cells express different isoforms of NOX, including the phagocytic NOX2 and the non-phagocytic NOX1, NOX4, DUOX1, and DUOX2 [56]. The inactivated form of hepatic stellate cells expresses low levels of regulatory and catalytic NOX components [58]. NOX became upregulated during hepatic injury and NOX4-related ROS production is a major driver for hepatic stellate cell activation and proliferation [50,59,60]. NOX4 deficiency significantly reduced ROS production and the expression of fibrogenic markers in hepatic stellate cells [61,62]. Similarly, the use of siRNA against NOX4 reduced hepatic stellate cell activation as well as the fibrotic phenotype of myofibroblast may be reversed, reducing NOX4 activity [51,56]. NOX1 and NOX2 also play a role in hepatic stellate cell activation, as shown by in vivo studies [50,52].

Among the factors contributing to the NOX-related hepatic stellate cell activation, the platelet-derived growth factor (PDGF) is a major one acting through the phosphorylation of the redox-sensitive MAPK p38 and through the activation of Na^+^/K^+^ exchanger peroxisome proliferator-activated receptor β [63]. Angiotensin II is another key effector peptide with pro-fibrogenic roles (e.g., proliferation, migration, collagen synthesis) on hepatic stellate cells in response to NOX-derived ROS [64]. Binding to the specific receptor (AT1) in hepatic stellate cells, angiotensin II induces the phosphorylation of p47phox [64]. These data are confirmed by the antifibrotic activity—through NOX reduction—of angiotensin-converting enzyme 2 [65]. Furthermore, TGF-β1 promotes hepatic stellate cell activation and there is reciprocal feedback between NOX and TGF-β1 in favor of fibrogenesis [66,67]. Further mechanisms of hepatic stellate cell activation include the phagocytosis of hepatocytes-derived apoptotic antibodies by hepatic stellate cells, leptin production—an adipocyte-derived hormone—and cannabinoid receptor 1 upregulation during liver damage [52,68].

### 3.2. Hepatocytes

As for the hepatic stellate cells, hepatocytes express both the phagocytic NOX2 and the non-phagocytic NOX1, NOX4, DUOX1, and DUOX2 isoforms [56]. More specifically, NOX4 expression is mediated by TGF-β, and NOX4 activity shares a relevant role in TGF-β-mediated hepatocyte apoptosis in both human and animal models through specific mechanisms (e.g., increasing the levels of pro-apoptotic proteins) [69,70,71]. NOX4 also promotes death ligand-induced hepatocyte apoptosis [56,61]. Even if hepatocytes express all components of NOX1 and NOX2, the mechanisms for their activation and their role in this kind of hepatic cells remain poorly understood [56].

### 3.3. Kupffer Cells

Kupffer cells are strategically located in the liver sinusoids and represent almost 80% of all macrophages in the body. They are responsible for the clearance of exogenous materials and the identification of endogenous molecular signals deriving from disrupted homeostasis pathways [72]. Once activated, Kupffer cells release biochemically active molecules, recruit non-resident cells (e.g., neutrophils, natural killer T lymphocytes) through the expression of adhesion molecules, eliminate detrimental particles, and present antigens to attract cytotoxic and regulatory T cells [72].

Kupffer cells mainly express the phagocytic NOX2 isoform that shares defensive activity against the bacterial products [56,73]. In this regard, it appears that lipopolysaccharide acts on Kupffer cells, activating NOX2 and stimulating NF-kB and pro-inflammatory cytokines production [73]. It has been shown that NOX2 expression in Kupffer cells is activated by several stimuli (e.g., TNF-α and alcohol metabolites) and was capable of indirectly activating the hepatic stellate cells through a paracrine mechanism [64,73,74]. Of note, ROS-mediated liver damage appears reduced after alcohol or diethylnitrosamine administration in NOX2-deficient mice [75,76].

A recent study showed that NOX4 also promotes an inflammatory response in Kupffer cells, activating the NLRP3 inflammasome through ROS production [77].

### 3.4. Sinusoidal Endothelial Cells

Liver endothelium, mostly represented by sinusoidal endothelial cells, has the role of a physical barrier that regulates molecular exchanges between the liver parenchyma and the circulation and is involved in several pathophysiological pathways, including vascular tone regulation and angiogenesis, hepatic immune response and inflammation, and metabolic homeostasis [78]. Sinusoidal endothelial cells mainly express NOX1, NOX2, and NOX4, even if at a lower rate than hepatic stellate cells and hepatocytes [56]. The upregulation of these NOX isoforms contributes to liver damage [78]. In response to NOX4 activity, sinusoidal endothelial cells release hepatocyte growth factors altering hepatocyte engraftment and hepatic cell regeneration [79]. Similarly, the activity of NOX2 affects liver capillarization and may play a central role in vessel remodeling [80]. Lastly, the up-regulation of NOX1 in sinusoidal endothelial cells related to a high-fat diet may promote cellular injury and impaired hepatic microcirculation through the reduced bioavailability of nitric oxide [46].

## 4. NOX in MASLD Development and Progression to MASH

The impact of different NOX isoforms on the development of MASLD and its progression to MASH was addressed in several experimental and clinical studies (Table 1) [81,82].

### 4.1. Experimental Studies

Several experimental studies have been performed during the last years to evaluate the role of NOX isoforms in MASLD development and its progression to MASH and liver fibrosis by enhancing oxidative stress-related liver damage [81,82,101]. The prevention of MASLD development in obese mice or in mice on a high-fat diet by the use of antioxidants or antiperoxynitrites also suggests a role for the nitro-oxidative stress caused by a family of molecules derived from nitric oxide—reactive nitrogen species—in the pathogenesis of these liver diseases [102,103,104].

Specifically, Larion and Colleagues recently reported that in obese mice, NOX1 deletion significantly attenuates hepatic oxidative stress and steatosis by reducing superoxide levels and modulating the insulin signal [83]. An up-regulation of the NOX1 was also observed in the liver of mice on a high-fat diet with cholesterol, and NOX1-deficient mice had significantly attenuated levels of serum alanine aminotransferase and hepatic cleaved caspase-3 that is related to apoptosis, fibrogenesis, and fibrosis [46,105]. Similarly, mice on a high-fat diet showed reduced levels of malondialdehyde and increased activity of superoxide dismutase in both plasma and liver tissue, consistent with a down-regulation of the liver expression of gp91phox subunit of NOX [89]. This evidence suggests the role of NOX2 in metabolic dysfunction and MASLD development [89,95].

García-Ruiz and Collaborators [45] also investigated the role of NOX in the pathogenesis of oxidative phosphorylation (OXPHOS) dysfunction of mice on a high-fat diet and concluded that NADPH deficiency protects mice from developing high-fat diet-induced OXPHOS dysfunction and MASH. In this experiments, normal OXPHOS activity, subunits and assembly of subunits into OXPHOS complexes, as well as mild steatosis without MASH lesions have been found in the liver of NOX^(−/−)^ mice on a high-fat diet [45]. Conversely, Greatorex and Colleagues reported that the NOX4 deletion in hepatocytes of obese mice on a high-fat diet leads to liver oxidative damage, inflammation, and recruitment of T cells, while the overexpression of NOX4 attenuates MASH and fibrosis development in mice fed a MASH-promoting diet [85].

Inflammatory response and oxidative stress are two known key players during MASLD progression to MASH [106] by altering hepatic lipid metabolism, exacerbating insulin resistance, inducing ROS generation, modulating the proinflammatory response, and developing fibrosis [107]. In fact, the presence of MASH-associated inflammation identifies MASLD patients at a higher risk of fibrosis and disease progression [107]. On the basis that a strong inflammatory phase occurs in MASLD progression to MASH, a rodent model of early steatohepatitis showed a significant increase in the co-localization of gp91 (membrane subunit) and p47phox (cytoplasmic subunit) [93]. Furthermore, Kupffer cells showed a significant increase in NOX activation in response to both leptin and lipopolysaccharides [93]. In this setting, NOX results in the production of ROS and inactivation of the nuclear factor (NF)-B pathway, which leads to a sustained proinflammatory and pro-fibrotic response. The down-regulation of NOX2 and the normalization of mitochondrial biogenesis and dynamics further decreased the expression of proinflammatory cytokines (i.e., TNFα, interleukin—IL—6, and IL1β) and increased the antioxidant capacity and expression of antioxidant enzymes (e.g., catalase, superoxide dismutase, glutathione peroxidase) [84]. In vitro experiments on hepatocytes confirmed these data. NOX2 overexpression increased cell apoptosis, ROS production, and inflammatory molecule levels (e.g., TNF-α, iIL1β, and IL6) [86]. Furthermore, the reduction in activated NF-κB of p47 phox knockout mice on a high-fat diet confirmed the role of NOX in this inflammatory pathway [94]. The attenuated release of proinflammatory cytokines and stellate cell activation in the MASLD mice model lacking the p47phox gene further supports the involvement of NOX in liver disease progression [86].

NOX2-mediated redox signaling can also activate micro RNAs [108]. Previous studies have shown the role of micro RNAs (miR21) in several types of chronic liver diseases. Expression of hepatic miR-21, one of the most up-regulated microRNAs in MASLD [86], is increased in animal models and in patients with MASLD/MASH and is able to promote fibrogenesis [109,110,111]. Specifically, treating the leptin-primed immortalized Kupffer cells—a mimicked model for an MASLD condition—with the selective NOX inhibitor apocynin significantly decreased CD68 and miR-21 [112]. These data provide evidence for the role of NOX2-dependent ROS in miR21-induced Kupffer cell activation and stellate cell pathology [92].

Knowing that the incidence of MASH strikingly rises with age and significantly contributes to elder mortality, some Authors focused their experiments on the age-related pathways responsible for accelerated fibrosis progression [113]. In this context, NADPH-derived oxidative stress has a key role in the progression of MASH and has been linked to aging pathways in several organs [114]. In the liver, the increased oxidative stress and accelerated fibrosis of aging are modulated by direct activation of the phagocytic NOX2 in hepatocytes through the p52Shc binding and the activation of the p47phox subunit [91].

### 4.2. Human Studies

The NOX activation would seem associated with liver damage in MASLD and MASH patients. Among the putative mechanisms accounting for liver disease, NOX isoforms could play an important role. This aspect has been deduced by the evaluation of NOX activation in patients with MASLD and MASH. In particular, many studies evaluated patients’ serum levels of soluble NOX2-derived peptide, which reflects NOX2 activation by representing the extramembrane portion of NOX2 released after enzyme activation [115].

MASLD patients showed lower levels of eicosanoids (e.g., isoprostanes) as well as of alanine aminotransferase compared to those without MASLD [96,100]. Of note, urinary 8-iso-PGF2α and serum soluble NOX2-derived peptide levels increased with the severity of liver steatosis at ultrasound evaluation [87]. These data were also found in children with MASLD showing an over-activation of NOX2 compared to the controls and a significant association of NOX2 with the degree of liver damage [98].

Furthermore, a randomized cross-over study in 19 patients with MASH and 19 patients with simple fatty liver steatosis highlighted higher NOX2 activity and isoprostanes levels with a significant gradient between fatty liver steatosis and MASH. The progressive increase in serum isoprostanes and NOX2 activity from patients with simple steatosis and patients with MASH was consistent with the “two-hit” theory [116,117]. According to this theory, after the “first hit” (liver steatosis), a “second hit”—including increased oxidative stress [35]—is required to develop MASH, confirming the association between the degree of liver damage and NOX2-derived oxidative stress [117]. NOX1-derived ROS production may further contribute to the progression of MASLD. A significant increase in NOX1 mRNA was observed in MASH patients compared with those from healthy controls [46]. In liver samples, the level of NOX1 tended to be higher in MASH patients [46].

Finally, it is important to underline that MASLD is characterized by different degrees of development and progression among individuals. The reason for these diversities is not fully known; however, environmental influences, including eating habits, intestinal microbiota, and multiple genetic factors, may have an influence. Many polymorphisms have been described that influence gene expression, including the gene encoding NOX4. In this regard, there were associations between the presence of polymorphisms for NOX4 and higher levels of alanine transferase in the MAFLD population, and higher levels of triglyceride and lower levels of high-density lipoprotein in MASH patients [97].

## 5. Perspectives and Conclusions

As oxidative stress and NOX-related ROS production play a central role in the pathophysiology of MASLD, careful regulation of the redox balance is essential to maintain lipid homeostasis. Dietary interventions aimed at improving the antioxidant status and restoring a “healthy” lipid profile surely represent a relevant part of the therapeutic management of patients with MASLD. Recent experimental studies also supported the potential beneficial effect of some antioxidants (e.g., vitamin E, vitamin C, metformin) and probiotics (e.g., Vinpo and Lactobacillus) in MASLD [11,118]. Knowing that specific vitamin deficiency elicits oxidative stress and systemic inflammation, a proper supply may protect against liver cell damage [119]. It has been demonstrated that vitamin E and C supplementation reduced the average levels of liver functionality tests in MASLD and liver fibrosis in MASH [119]. Further studies will be needed to evaluate whether vitamin supplementation has a beneficial effect on MASLD and MASH through NOX-related mechanisms.

Targeting NOXs as a therapeutic approach to reduce the development and progression of MASLD is an interesting topic. However, it should be acknowledged that the inhibition of NOX activity, and especially NOX2, could have deleterious consequences for the innate immune response [120]. Knowing the above, direct inhibition of other specific NOX isoforms that act in the liver (e.g., NOX1 or NOX4) may reduce the chance of side effects. Even if finding selective NOX inhibitors is hampered by the high degree of structural and catalytic homology within various NOX isoforms, some molecules that selectively inhibit NOX1 and NOX4 have been identified (e.g., GKT136901 and GKT137831) [121,122]. In contrast to gene deletion, these inhibitors do not completely suppress ROS production and appear well-tolerated in their use for the prevention of disease progression in a range of models of chronic inflammatory and fibrotic conditions [121]. Interestingly, GKT137831 reduced ROS production, liver fibrosis, and hepatocyte apoptosis as well as messenger RNA expression and NOX genes in mice models [44,60,61]. Additional studies will be needed to expand the knowledge on the topic and to identify further molecules and therapeutic approaches that may improve patients’ management and prognosis through regulation of the oxidative status.

## Figures and Tables

**Table 1:** Experimental and Human Studies evaluating the role of NOX in MASLD and MASH.

References	NOX Isoform	Disease Status	Experimental Model	Main Outcomes in Association with NOXs
*Experimental Studies*				
Larion S. et al., 2024 [83]	NOX1	MASLD	NOX1 knockout mice: lean and db/db	↑ Superoxide ↑ Insulin signaling ↑ Fat accumulation in the liver
Tanaka M. et al., 2024 [84]	NOX2	MASH-derived sarcopenia	MCD-fed mice steatohepatitis and skeletal muscle atrophy model	NOX2 down-regulation induces: ↓ Proinflammatory cytokines (i.e., TNFα, IL6, and IL1β) ↑ Antioxidant capacity ↑ Antioxidant enzymes ↑ Hepatic and plasma IGF-1
Greatorex S. et al., 2024 [85]	NOX4	MASLD/MASH	1. Hepatocyte NOX4 deletion in HFD obese mice: develop steatosis, but not MASH 2. Hepatocyte NOX4 over-expression in mice fed a MASH-promoting diet	1. ↑ Hepatic oxidative damage ↑ Inflammation ↑ T cell recruitment 2. ↓ MASH and fibrosis
Ji J. et al., 2022 [86]	NOX2	MASLD	HFD-fed mice	NOX2 over- expression induces: ↓ liver function ↑ ROS levels ↑ TNF-α, IL-1β and IL-6
Grossini E., et al., 2021 [87]	NOX2	MASLD	Human hepatocellular carcinoma cells (Huh7.5) treated with plasma from: 1. 12 MAFLD patients 2. 12 Healthy subjects	Plasma of MAFLD patients induced: ↑ H_2_O_2_ ↑ Mitochondrial ROS ↓ Mitochondrial membrane potential ↑ Triglycerides ↑ NF-kB ↑ NOX2
Zou Y. et al., 2021 [88]	NOX4	MASLD	HFD-fed Zebrafish	↑ NOX4 ↑ ROS ↓ MDA
Bunbupha S. et al., 2021 [89]	NOX2	MASLD	HFD-fed rats down-regulation of liver NOX2	In plasma and hepatic tissue ↓ MDA ↑ SOD activity
Sarkar S. et al., 2020 [90]	NOX2	MASLD	p47phox knockout CD-HFD-fed mice	↓ Collagen protein (fibrosis) in intestine
Jiang JX et al., 2020 [91]	NOX1 NOX2 NOX4	MASH	Old-Fast food diet mice	↑ NOX2 NOX4 and NOX1 not induced
Albadrani M. et al., 2019 [92]	NOX2	MASLD/MASH	1. p47phox knockout HFD-fed mice 2. Leptin-primed immortalized Kupffer cells (SV40)	1. ↓ TNF-α ↓ Stellate cell activation ↓ NOX2-derived peroxynitrite 2. SV40 cells treated with apocynin: ↓ NOX2-derived peroxynitrite ↓ Kupffer cell activation ↓stellate cell pathology
Matsumoto M., 2018 [46]	NOX1	MASLD	1. HFD-fed and HFC-fed mice 2. NOX1-knockout mice	1. ↑ NOX1 expression ↑ Hepatic cleaved-C3 2. ↓ Hepatic cleaved-C3 ↓ Peroxynitrite injury in hepatic sinusoids ↓ Nitrotyrosine adducts
García-Ruiz I. et al., 2016 [45]	NOX2	MASH	HFD-fed NOX1- knockout mice	- Mild steatosis but no MASH lesions - Normal OXPHOS activity and subunits
Dattaroy D. et al., 2015 [93]	Not specified	MASH	Steatohepatitic injury mice HFD-fed mice Kupffer Cells culture	↑ gp91/p47phox colocalization ↑ Peroxynitrite formation
Dattaroy D. et al., 2014 [94]	Not specified	MASH	1. HFD-fed mice 2. p47phox knockout CD-HFD-fed mice	1. ↑ Oxidative stress ↑ p47phox expression ↑ NF-κB activation 2. ↓ NF-κB activation ↓ Fibrogenesis
Carmiel-Haggai M. et al., 2004 [95]	Not specified	MASLD	Obese HFD-fed rats	↑ NOX activity ↑ Lipid peroxidation ↑ Protein carbonyl formation ↓ Antioxidant defense
***Human Studies***				
Baratta F. et al., 2020 [96]	NOX2	MASLD	MASLD patients (*n* = 193) Cardio-metabolic patients without MASLD (*n* = 45)	↑ sNOX2-dp
Rabelo F. et al., 2018 [97]	NOX4	MASLD	MASLD patients (*n* = 207)	Association between SNPs in the NOX4 gene and alanine transferase
Loffredo L. et al., 2019 [98]	NOX2	MASLD	MASLD children (*n* = 67) Controls (*n* = 73)	↑ sNOX2-dp ↑ Isoprostanes ↑ Triglycerides ↑ HOMA-IR ↑ Fasting glucose and insulin Linear association between sNOX2-dp and degree of liver damage
Loffredo L. et al., 2017 [99]	NOX2	MASH	MASH (*n* = 19), Fatty liver disease (*n* = 19), controls (*n* = 19)	↑ sNOX2-dp ↑ Isoprostanes ↓ FMD ↓ NOx bioavailability
Matsumoto M et al., 2018 [46]	NOX1	MASH	MASH patients	↑ NOX1 expression in liver
Del Ben M. et al., 2014 [100]	NOX2	MASLD	Steatosis patients ith or without MASLD (*n* = 264)	↑ sNOX2-dp ↑ Urinary 8-iso-PGF2α Urinary 8-iso-PGF2α and sNOX2-dp increase with the liver steatosis severity

FMD, flow-mediated dilation; HFD, high-fat diet; MASH, metabolic dysfunction-associated steatohepatitis; MASLD, metabolic dysfunction-associated steatotic liver disease; NOX, nicotinamide adenine dinucleotide phosphate oxidases.
